# Topological correlations of structural and functional networks in patients with traumatic brain injury

**DOI:** 10.3389/fnhum.2013.00726

**Published:** 2013-11-05

**Authors:** Karen Caeyenberghs, Alexander Leemans, Inge Leunissen, Karla Michiels, Stephan P. Swinnen

**Affiliations:** ^1^Department of Physical Therapy and Motor Rehabilitation, Faculty of Medicine and Health sciences, University of GhentGhent, Belgium; ^2^Department of Movement and Sport Sciences, Faculty of Medicine and Health sciences, University of GhentGhent, Belgium; ^3^PROVIDI Lab, Image Sciences Institute, University Medical Center UtrechtUtrecht, Netherlands; ^4^Group Biomedical Sciences, Movement Control and Neuroplasticity Research Group, KU LeuvenLeuven, Belgium; ^5^Department of Physical Medicine and Rehabilitation, University Hospital, Leuven Campus PellenbergLeuven, Belgium

**Keywords:** functional connectivity, structural connectivity, brain networks, graph theoretical analysis, brain injury

## Abstract

Despite an increasing amount of specific correlation studies between structural and functional connectivity, there is still a need for combined studies, especially in pathological conditions. Impairments of brain white matter (WM) and diffuse axonal injuries are commonly suspected to be responsible for the disconnection hypothesis in traumatic brain injury (TBI) patients. Moreover, our previous research on TBI patients shows a strong relationship between abnormalities in topological organization of brain networks and behavioral deficits. In this study, we combined task-related functional connectivity (using event-related fMRI) with structural connectivity (derived from fiber tractography using diffusion MRI data) estimates in the same participants (17 adults with TBI and 16 controls), allowing for direct comparison between graph metrics of the different imaging modalities. Connectivity matrices were computed covering the switching motor network, which includes the basal ganglia, anterior cingulate cortex/supplementary motor area, and anterior insula/inferior frontal gyrus. The edges constituting this network consisted of the partial correlations between the fMRI time series from each node of the switching motor network. The interregional anatomical connections between the switching-related areas were determined using the fiber tractography results. We found that graph metrics and hubs obtained showed no agreement in both groups. The topological properties of brain functional networks could not be solely accounted for by the properties of the underlying structural networks. However, combining complementary information from both imaging modalities could improve accuracy in prediction of switching performance. Direct comparison between functional task-related and anatomical structural connectivity, presented here for the first time in TBI patients, links two powerful approaches to map the patterns of brain connectivity that may underlie behavioral deficits in brain-injured patients.

## Introduction

Many patients with traumatic brain injury (TBI) are faced with persistent cognitive deficits, including impairments in information processing speed, memory, and executive function, which limit recovery (Levin and Kraus, 1994; Miller, 2000; Godefroy, 2003). The clinical pathology underlying this poor cognitive outcome is traumatic axonal injury (TAI), which is characterized by widespread axonal damage due to shearing forces by acceleration, deceleration, or rotation of the brain. TAI disrupts the efficient functioning of brain networks, which consists of white matter (WM) tracts that connect brain regions As cognitive control depends on the coherent activity of widely distributed networks, it is important to examine the characteristics of the brain networks in TBI.

The area of graph theory is an established mathematical field and has proven a very effective and informative way to explore brain networks and human behavior (Bassett and Bullmore, 2009; Bullmore and Sporns, 2009) in health (e.g., Iturria-Medina et al., 2008; Li et al., 2009) and disease (for an overview, see Griffa et al., 2013). With graph theory, the brain can be represented in an abstract manner as a set of “nodes,” defined by anatomical regions across the cortex, and “edges,” which reflect connection properties between these nodes (e.g., Hagmann et al., 2008). While the node/edge characteristics are typically represented by “connectivity matrices,” a graph theoretical analysis (GTA) provides a novel way to explore topological and geometrical properties of brain networks, such as clustering coefficient, small worldness, efficiency, path length, connectivity degree, among others [for an in-depth discussion of these measures, see (Rubinov and Sporns, 2010)].

As TAI disrupts the connections of distributed brain networks, GTA has already offered insights into the dysfunction of these networks following TBI using different imaging modalities. For example, using fMRI-based GTAs (Caeyenberghs et al., 2012a), patients with TBI showed increased connectivity degree and strength, and higher values of local efficiency, compared with controls. On the other hand, diffusion MRI-based GTAs have shown reduced connectivity degree, longer average path lengths, and reduced network efficiency in brain-injured adults (Caeyenberghs et al., 2012b; Pandit et al., 2013) and children (Caeyenberghs et al., 2012c). These findings suggest that TBI affects the global organization of the brain network and support the notion of TBI as a “disconnection syndrome” from a network perspective (Guye et al., 2010).

However, most studies have used only one of the imaging modalities at a time. Greater effort should be focused on the integration of different modalities, since combining complementary information from the different imaging modalities may be more fruitful than using either one alone (for a review, see Damoiseaux and Greicius, 2009). It can be especially helpful in studying disease (e.g., Andrews-Hanna et al., 2007; Rocca et al., 2007; Lowe et al., 2008; Skudlarski et al., 2010; Palacios et al., 2012). For example, Lowe et al. (2008) found that in a cohort of 11 multiple sclerosis patients and 10 control subjects, mean FA of the transcallosal motor pathway, as derived from DTI, correlated positively with functional connectivity of the bilateral primary sensorimotor cortices, as measured with resting state fMRI.

In these studies, WM microstructural measures usually come in the form of single scores. However, it is important and informative to compare *equidimensional* structural and functional connectivity maps/matrices, that is, cases in which both structural and functional connectivity indices are available for the same pairs of regions-of-interest. Moreover, in order to extract relevant information from the brain's structure and function, it is necessary to validate them against different parameters of another framework, such as GTA. For example, Hagmann et al. (2008) and Honey et al. (2009) were, to the best of our knowledge, the first to use GTA to directly compare resting-state functional connectivity with structural connectivity. The authors found that the strength of resting-state functional connectivity correlated positively with structural connectivity strength in healthy participants. However, there is still a need for combined studies, especially in pathological conditions.

In this paper, we compared the graph metrics of task-related functional connectivity, using event-related fMRI, and structural connectivity, derived from fiber tractography using diffusion MRI data, computed in the same participants (17 adults with TBI and 16 controls). Our primary goal was to test the hypothesis that TBI patients would show a negative correlation between the two aspects of brain connectivity, i.e., TBI patients who exhibit more profound structural deficits (lower structural connectivity) would show higher functional connectivity (to compensate). Specifically, TAI might cause reorganization of functional connectivity and thus cause a negative association between functional and structural connectivity. This was predicted on the basis of relevant earlier work showing that patients with TBI who showed higher functional connectivity degree displayed lower switching task performance and more severe brain injury (Caeyenberghs et al., 2012a). Conversely, the results of our diffusion MRI based GTA's showed a decrease in global integration in structural networks in TBI patients (Caeyenberghs et al., 2012b,c). These earlier results suggested that the higher functional network cohesion in the TBI group may be directly related to a poorer neurobiological substrate, i.e. structural disconnection between brain areas or lower structural connectivity. Moreover, we sought to validate whether complementary structural and functional connectivity information can be combined to improve accuracy in prediction of behavioral deficits.

## Materials and methods

### Participants and MRI data acquisition

The present study included data from 17 adults with TBI and 16 controls. The TBI patients had sustained closed-head trauma due to traffic accident or sport injury that averaged 4 years 3 months prior to the study (*SD* = 2 years 5 months). The majority of patients sustained moderate to severe TBI as measured by the postresuscitation Glasgow Coma Scale (GCS, Teasdale and Jennett, 1974) (only available from 4 patients, *M* = 7.8, range = 4–12), the duration of loss of consciousness (30 min or more), the length of post-traumatic amnesia (>1 day), the anatomical features of the injury based on inspection by an expert neuroradiologist (see below), and the injury mechanism (traffic accidents and falls), or combinations thereof. Informed consent was obtained from each subject, and ethical approval was granted by the local ethics committee for biomedical research.

Diffusion tensor images (Figure 2B) were acquired with a Siemens 3 T Magnetom Trio MRI scanner (Siemens, Erlangen, Germany) using the following parameters: single shot spin-echo; slice thickness 2.9 mm; repetition time (TR) 7200 ms, echo time (TE) 81 ms, number of diffusion directions 64, diffusion weighting 1000 s/mm^2^, number of sagittal slices 56, in-plane resolution 2.2 × 2.2 mm^2^ with a field of view of 210 × 210 mm^2^.

Functional data (fMRI) (Figure 2A) were acquired with a descending gradient echo planar imaging (EPI) pulse sequence for T2^*^-weighted images (*TR* = 3000 ms, *TE* = 30 ms, flip angle = 90°, 50 oblique axial slices each 2.8 mm thick, inter-slice gap 0.028 mm, in-plane resolution 2.5 × 2.5 mm^2^, and matrix size of 80 × 80).

Finally, a high resolution T1-weighted structural image was acquired using magnetization prepared rapid gradient echo (MPRAGE; *TR* = 2300 ms, *TE* = 2.98 ms, 1 × 1 × 1.1 mm^3^ voxels, field of view (FOV): 240 × 256 mm^2^, 160 sagittal slices). These structural MRI scans were inspected and classified by an experienced neuro-radiologist (S.S.). Demographic and neurologic variables are provided in Table 1.

**Table 1:** **Summary of demographic and injury characteristics for the TBI group**.

**TBI patient no. age/gender/handedness**	**Age at injury**	**GCS score**	**Acute scan within 24 hours after injury lesion location/pathology**	**MRI scan at examination lesion location/pathology**
TBI 1 27.6/F/RH	25.2		TL contusion, (R) PL haemorrhage, (L) FL intraparenchymateus hemorrhagic contusion, subdural hematoma	Drain tract (R), (L) FL and TL contusion
TBI 2 22.9/F/RH	21.3		(R) FL haemorrhage, (L) FL/TL and (L) PL and (R) orbito-frontal cortex contusion	Drain tract (R), hemosiderin deposits (R) PL and (R) orbito-frontal cortex
TBI 3 22.5/M/RH	17.6		(L) FL shearing injuries, splenium and body corpus callosum contusion	(R) FL contusion
TBI 4 28.1/M/RH	18.6	12	FL contusion, (L) FL subdural hematoma, (L) TL and (R) PL haemorrhage	Drain tract (L), FL contusion
TBI 5 17.9/F/RH	12.9		Contusion (location not specified in available records)	–
TBI 6 34.6/M/RH	28.9		(R) amygdala and basal ganglia and (R) PL haemorrhage, (L) FL inflammatory changes	(L) TL contusion
TBI 7 16.8/M/RH	9.1	8	(L) TL and (L) FL punctiform and (R) mesencephalon contusion, (L) FL and (L) thalamus hemorrhagic injuries	Orbito-fronal cortex contusion, enlarged ventricles
TBI 8 33.8/M/RH	27.9			Drain tract (R), thalamus injury, corpus callosum shearing injuries, (R) FL and (L) inferior FL and (R) OL contusion
TBI 9 26.9/F/RH	23.9		FL injuries	Drain tract (L), PL and OL/PL and FL and (R) TL shearing injuries, slightly enlarged ventricles
TBI 10 22.3/M/RH	19.1		Contusion and DAI (location not specified in available records)	(L) thalamus and (L) TL and (L) orbito-frontal cortex and (L) FL and (R) FL and central sulcus shearing injuries
TBI 11 31.7/M/RH	29.6		(L) FL/TL haemorrhage and DAI, FL and TL/OL shearing injuries	TL and (R) orbito-frontal cortex and (R) inferior FL contusion, corpus callosum degeneration, asymmetric ventricles, (L) PL shearing injury
TBI 12 16.7/M/RH	14.5		Enlarged (R) lateral ventricle, (R) hematoma occipital horn lateral ventricle, hyperdensity (L) thalamus and PL/TL, (LH) shearing injuries	Drain tract (R), (L) corpus callosum and thalamus and (R) PL and (L) FL and (R) TL shearing injuries, occipital horn lateral ventricle asymmetrically enlarged
TBI 13 28.1/M/RH	18.4		Hemosiderin deposits corpus callosum, DAI, ischemic injury (L) occipital horn of lateral ventricle	Drain tract (R), (R) periventricular white matter FL and thalamus injuries, corpus callosum degeneration
TBI 14 27.9/M/RH	24.9	7	(L) thalamus and (L) periventricular and corpus callosum and brainstem and TL shearing injuries	Drain tract, (L) thalamus and corpus callosum and (L) TL shearing injuries
TBI 15 30.9/M/RH	28.3		Lesion and location not specified in available records	Drain tract (R), (L) inferior TL contusion, (L) anterior cingulate and (R) FL and central sulcus shearing injuries
TBI 16 24.1/M/RH	21.8		(L) FL hematoma, FL intraparenchymal hemorrhage, subarachnoidal bleeding	Drain tract (R), orbito-frontal cortex and (L) cerebellum contusion
TBI 17 20.6/F/RH	18.1	4	Diffuse axonal injuries, (L) FL/TL/PL subdural hematoma, FL contusion, injuries corpus callosum	FL and (R) PL contusion, orbito-frontal cortex shearing injuries, enlarged ventricles

Anatomy codes: RH, right hemisphere; LH, left hemisphere; FL, frontal lobe; TL, temporal lobe; PL, parietal lobe; OL, occipital lobe; R, right; L, left. Other codes: TBI, traumatic brain injury; MRI, magnetic resonance imaging; RH, right-handed; LH, left-handed; M, male; F, female, GCS, Glasgow Coma Scale score.

### Behavioral testing

**Figure 1:**
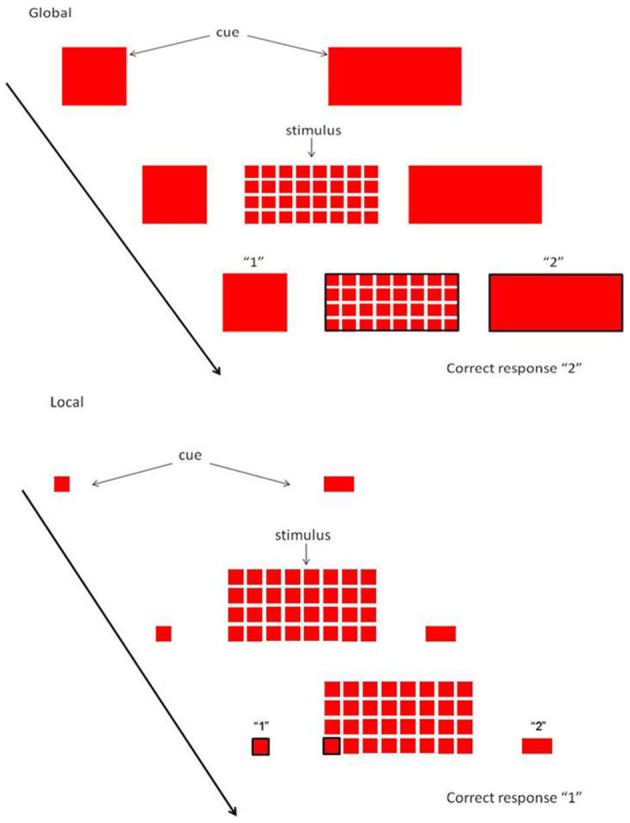
**Local Global Task.** Each trial started with a cue, indicating whether attention had to be paid to the global or local level. When the stimulus appeared, subjects had to rapidly decide whether the relevant level consisted of squares or rectangles.

Assessment of executive function was performed using the Local Global Task (LGT). Participants performed the LGT (derived from Miyake et al., 2000) with their right hand. The target stimulus (as shown in Figure 1) consisted of a “global” square or rectangle, composed of much smaller “local” squares or rectangles. Each trial began with the presentation of a prime cue, indicating to which dimension to attend. The global dimension was cued by a big square, to the left of the stimulus, and a big rectangle to the right. For the local dimension the same square and rectangle appeared, only smaller. After a random cue-target interval of 400–600 ms, the target stimulus was presented. Both the cue and the target stimulus remained on the screen until a participant responded, or until 2500 ms had elapsed. Participants were required to identify the relevant target stimulus dimension and press one key with their index finger for squares and another with their middle finger for rectangles (Figure 1). The interval between a response and the presentation of the next trial varied randomly between 900 and 1100 ms. The experiment was comprised of two unidimensional blocks, and one switch block. In the unidimensional blocks, participants attended to either the global cues or the local cues. The order was counterbalanced across participants. In the third switch block, the target stimulus dimension alternated every other trial (i.e., two “local” trials, followed by two “global” trials, etc.). When the prime cues changed, the participants had to switch from responding to the local dimension to the global dimensions of the target stimulus, and vice versa. A short amount of practice was given to ensure the instructions were understood (4 trials for each unidimensional block, and 8-16 trials for the switch block). The experiment consisted of 24 trials in each pure block, and 49 trials in the switch block. Variables of interest were RT and accuracy on repetition trials and on switch trials, and switch cost (=RT switch trial—RT repetition trial). The whole task took about 15 min.

### Preprocessing

In preparation for the definition of the nodes, the fMRI time series were passed through several preprocessing steps using the SPM 5 software package (Wellcome Department of Imaging Neuroscience, University College, London) implemented in MATLAB 7.7 (Mathworks, Sherborn, MA). The first three functional images of each subject's data set were discarded to allow for T1 equilibration. The remaining images were spatially realigned to the first image in the time series, then corrected for differences in slice acquisition time by temporal interpolation to the middle slice (reference slice = 25). Functional images were spatially coregistered to the anatomical image, and normalized using a combination of cost function masking and a unified segmentation procedure (Ashburner and Friston, 2005; Crinion et al., 2007). Finally, the normalized functional images were smoothed with an isotropic 10 mm FWHM Gaussian kernel.

The DTI data were analyzed and processed in ExploreDTI (Leemans et al., 2009; Jones and Leemans, 2011), as previously described in detail (Caeyenberghs et al., 2010a,b, 2011). In summary, for each data set the diffusion-weighted MRI images were corrected for subject motion and eddy-current induced geometrical distortions correction (Leemans and Jones, 2009). During this processing step, we adjusted the B-matrices with the appropriate reorientations and included the required signal intensity modulation with the Jacobian determinant of the spatial transformation (Leemans and Jones, 2009; Jones and Cercignani, 2010). The diffusion tensor was estimated using a non-linear regression procedure (Veraart et al., 2012) from which the diffusion metrics (e.g., fractional anisotropy—FA) were computed for further analysis (Basser and Pierpaoli, 1996).

### Subject motion

From the realigned fMRI data, it was verified that no subject had head movement larger than 2 mm in any direction during any of the functional runs (translational movements: TBI: mean = 0.55 mm, range = 0.19–1.09 mm; Controls: mean = 0.38 mm, range = 0.18–0.58 mm; rotational motions: TBI: mean = 0.53°, range = 0.23–1.02°; Controls: mean = 0.38°, range = 0.23–0.61°).

Translational motions did not exceed 1 voxel for the DTI data (TBI: mean = 0.95 mm, range = 0.40–1.44 mm; Controls: mean = 0.73 mm, range = 0.37–1.19 mm). Rotations of the DTI data were on average 0.81° and ranged between 0.31 and 1.31° for the TBI group; in the control group rotations were on average 0.68°, ranging between 0.22 and 1.25°.

### White matter tractography

**Figure 2:**
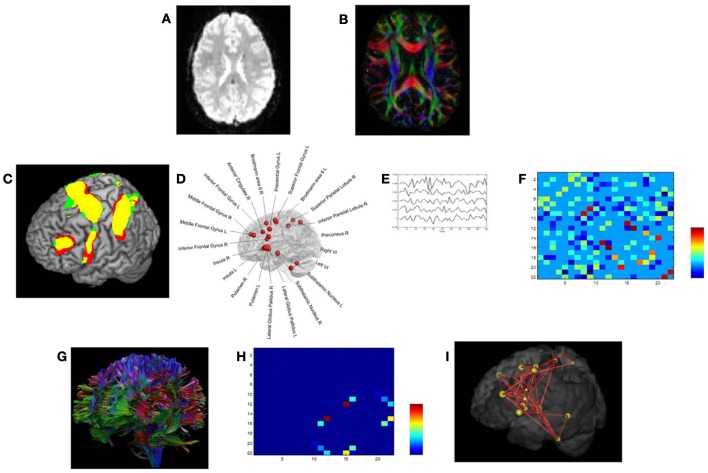
**Structural and functional brain connectivity was examined using graph theory through the following steps.** First, we acquired task-related fMRI data **(A)** and DTI data **(B)** in the same participants. **(C,D)** We defined the network nodes as fMRI activation foci. A sphere with radius (of 10 mm) was placed around the MNI coordinates of each ROI's activation peak. **(E)** For each subject, the average time series for each ROI was extracted for the Switch > Continue condition in an event-related fMRI design (Coxon et al., 2010; Leunissen et al., 2013a). **(F)** Based on the average time series data, matrices of partial correlations were then calculated, quantifying the unique functional relationships between each pair of ROIs (Caeyenberghs et al., 2012a). **(G)** Next, using a deterministic tractography approach, the number of white matter trajectories between each pair of regions of the switching motor network was determined. **(H)** This value became the edge weight in the structural connectivity matrix. **(I)** Finally, from the resulting brain networks, graph metrics, including connectivity degree, connection strength, regional efficiency, and betweenness centrality, were computed.

The interregional anatomical connections between the switching-related areas were determined using the fiber tractography results as obtained with *ExploreDTI* (Figure 2G) (Basser et al., 2000; Leemans et al., 2009). These fiber pathways were generated by starting seed points sampled uniformly throughout the data at 2 mm isotropic resolution. Trajectory propagation was terminated if FA < 0.2 or if the angle between consecutive steps exceeded 45°. The step size was set at 1 mm.

### Definition of the nodes and edges

Our network of particular interest was the switching motor network. This group of 22 brain regions (see Figure 2D), encompassing the medial frontal cortex (SMA: pre-SMA and SMA-proper), anterior cingulate cortex, bilateral dorso-lateral prefrontal cortex (DLPFC), inferior frontal cortex (BA44), basal ganglia (globus pallidus, putamen, subthalamic nucleus region), bilateral cerebellum (lobule VI), right precuneus, left premotor cortex (dorsal and ventral), bilateral insula, and right superior and right inferior parietal lobules was active during Switch > Continue in an event-related fMRI design (Figure 2C, Coxon et al., 2010; Leunissen et al., 2013b).

As a functional measure (Figures 2E,F) we calculated, in each of the 31 subjects, the partial correlations between each pair of ROI's mean time series, filtering out the effects of the remaining 20 brain regions (for details, see Caeyenberghs et al., 2012a). The structural measure for each subject was the number of WM trajectories connecting the ROIs (Figure 2H) (Gong et al., 2009; Lo et al., 2010; Hagmann et al., 2010; van den Heuvel et al., 2010). Self-connections of nodes were not included in the analyses.

A weighted graph approach was used, with the partial correlation representing a proxy measure for the strength of functional connectivity, and the number of WM trajectories as a weight value for structural connectivity. In addition to the weighted connectivity matrices, we also calculated unweighted binary matrices, in which the weighting was omitted from the analysis. For each individual dataset, all non-zero weights were set to one and to zero otherwise (van den Heuvel et al., 2010). Thus, for each participant, there were four different kinds of networks (weighted structural, binary structural, weighted functional, binary functional), each of which was represented by a symmetric 22 × 22 matrix.

Within the main analysis, the weights of the connections for structural connectivity were determined by means of the number of WM trajectories. An alternative measure for connectivity strength could be the level of FA, as FA values are regularly used as a measure of WM microstructural organization and as a marker for WM abnormalities in patient studies (Beaulieu et al., 2005; Mori et al., 2007; Caeyenberghs et al., 2010a,b, 2011). Therefore, an additional analysis was performed in which FA values were used as a measure of connectivity strength. In this additional analysis, a similar weighted graph analysis was performed but this time the weights of the connections were determined by means of the FA values of the interconnecting WM connections rather than the number of tracts. Similar to the analysis using the number of WM trajectories, overall graph organizational properties (efficiency, strength, and betweenness centrality) were computed and compared between groups.

### Graph theory analysis

The properties of the switching network were investigated at the global and regional (nodal) levels using the Brain Connectivity Toolbox (Rubinov and Sporns, 2010; https://sites.google.com/site/bctnet/). The equations to calculate each of these measures can be found in Rubinov and Sporns (2010). We only provide brief, formal definitions for each of the network properties used in this study: connectivity degree, connection strength, regional efficiency, and betweenness centrality (Figure 2I). Node degree is the number of links connected to the node. Node strength is the sum of weights of links connected to the node. The local efficiency is the average inverse shortest path length in the network (global efficiency) computed on node neighborhoods. Betweenness centrality is the fraction of all of the shortest paths in a network that contain a given node, with higher numbers indicating participation in a large number of shortest paths. The nodes with the largest betweenness centrality were considered to be pivotal nodes (i.e., hubs) in the network. Specifically, nodes were identified as hubs in the network if the values of nodal betweenness were 2 SDs greater than the average betweenness centrality of the network.

### Statistical analysis

Demographic data, including age and handedness, were examined for between-group differences with *t*-tests. Analysis of the reaction times of the LGT were subjected to a repeated-measures ANOVA with factors Group (TBI, controls), Cue condition (Global, Local), and Switch condition (Switch, Repeat). Significant main and interaction effects were further explored by post hoc tests using Tukey correction. For the switch cost and accuracy rate of the LGT, two-sample *t*-tests were performed for comparing the TBI group with the age matched control group. Moreover, controls and patient subgroups with better and poorer switching skills (based on a median-split of the accuracy rate) were separated and used for further analyses (see below). Between-group differences in the functional and structural connectivity were evaluated using two-sample *t*-tests, with graph measures (i.e., degree, strength, local efficiency, and betweenness centrality) from each approach (DTI or fMRI) examined as dependent variables. Pearson correlations were used to determine the association between structural and functional connectivity. Finally, separate discriminant function analyses (based on a median-split of the accuracy rate) were performed to identify the predictive accuracy of (1) the model with degree of the structural connectivity, (2) the model with degree of the functional connectivity, and (3) the model with the combination of the two imaging modalities. These analyses allowed us to discern the specific potential of the modalities (structural, functional, or combination) to distinguish between both groups. Discriminatory power of the models was quantified by the resultant sensitivity, specificity, overall classification accuracy and the Wilks' lambda statistic (1 = no discriminatory power; 0 = perfect discriminatory power).

## Results

### Demographic characteristics

Demographic features and clinical characteristics for the patients enrolled in this study are shown in Table 2. No significant difference in age were found between controls (*M* = 24.5 years, *SD* = 1.5 years) and patients (*M* = 24.9 years, *SD* = 5.8 years), [*t*_(31)_ = −0.30, *p* = 0.77]. Controls and patients did not differ by handedness, as defined by the Edinburgh Handedness Inventory (Oldfield, 1971) (laterality quotient: TBI: mean = 81, range = 22–100; control: mean = 92; range = 60–100).

**Table 2:** **Graph metrics of the switching network of both imaging modalities, mean, and standard error for both groups**.

**Graph metric**	**TBI group (*N* = 17)**		**control group (*N* = 16)**			
	**Mean**	***SE***	**Mean**	***SE***	***T***	***p***
**STRUCTURAL CONNECTIVITY (DTI)**						
Strength	1.861	0.097	1.818	0.072	−0.349	0.729
Degree	0.089	0.005	0.087	0.003	−0.353	0.727
Efficiency	0.137	0.01	0.131	0.009	−0.427	0.673
Betweenness centrality	3.561	0.55	3.744	0.818	0.188	0.852
**FUNCTIONAL CONNECTIVITY (FMRI)**						
Strength	**1.593**	**0.035**	**1.469**	**0.044**	**–2.239**	0.032^*^
Degree	5.861	0.16	5.449	0.195	−1.645	0.11
Efficiency	0.577	0.007	0.559	0.009	−1.549	0.132
Betweenness centrality	19.877	0.754	21.612	1.021	1.384	0.176

*Results of the two-sample-t-tests, bold values indicate significant results (p < 0.05)*.

* *p < 0.05*.

### Differences in behavioral performance on the local global task (LGT)

**Figure 3:**
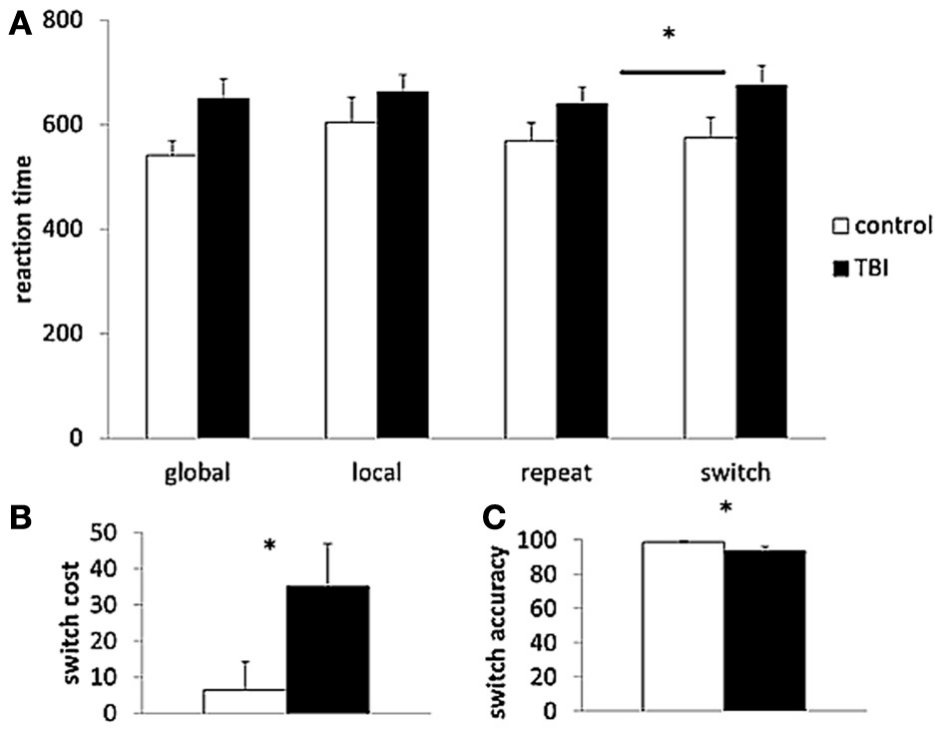
**Behavioral task performance. (A)** Reaction time of the different trial types (global, local, repeat, switch); **(B)** switch cost; and **(C)** accuracy rate of the Local Global Task. TBI, black bars; control, white bars. ^*^p < 0.05 for the TBI group compared to controls; TBI, traumatic brain injury.

For reaction times of the local and global trials in the LGT, there was only a significant main effect of Cue condition [*F*_(1, 29)_ = 5.99, *p* < 0.05], indicating that global level information (597 ± 23 ms) was processed faster than information of local trials (634 ± 29 ms). For reaction times of the repeat and switch trials, there was a significant main effect of Switch condition [*F*_(1, 29)_ = 8.63, *p* < 0.01], with longer reaction times for the switching trials. Moreover, there was a significant interaction effect between the two factors Switch condition and group [*F*_(1, 29)_ = 4.11, *p* < 0.05] (Figure 3A). *Post hoc* (Tukey) testing revealed only a significant difference between the switch (676 ± 37 ms) and repeat (641 ± 32 ms) trials within the TBI group. The mean accuracy rate (of the switch trials) and switch cost (switch reaction time – repeat reaction time) differed significantly between the TBI patients and the controls, accuracy: [*t*_(29)_ = 2.11, *p* < 0.05, switch cost: *t*_(29)_ = −2.03, *p* < 0.05] with the lower accuracy scores and higher switch cost in the TBI subjects indicating poorer switching performance than in the controls (see Figures 3B,C).

### Group differences in connectivity

While the graph metrics of the structural connectivity were basically identical between the groups (all *p*'s > 0.05), the TBI group consistently showed the tighter functional connectivity as compared to controls, which manifested in a higher connection strength [*t*_(31)_ = −2.24, *p* < 0.05] between the network nodes of the switching network (see Table 2).

### Associations between structural and functional connectivity

**Figure 4:**
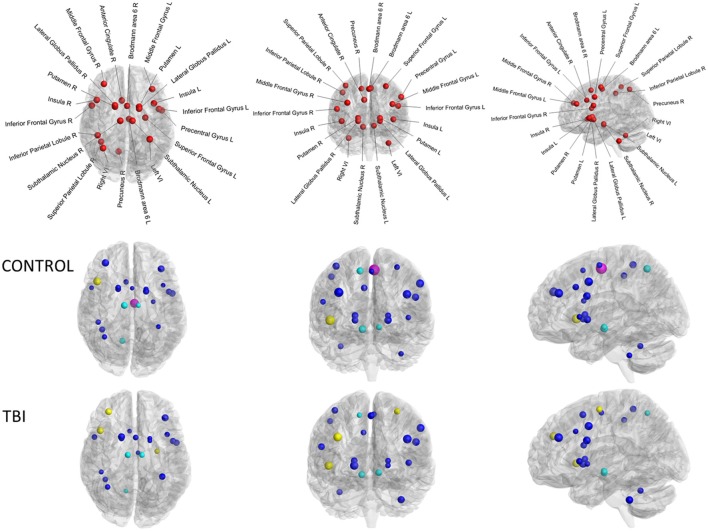
**Connectivity degree of the diffusion MRI versus connectivity degree of the task-related fMRI of the 22 brain regions. Upper** panel controls, **lower** panel TBI patients. Size of the ROIs (spheres) represents absolute value of the correlation coefficient. The colors of the nodes refer to: magenta significant correlation, blue not significant. Moreover, yellow spheres indicate hubs of the functional connectivity, cyan spheres represent hubs of the structural connectivity.

Whilst each of the graph measures emphasizes a different facet of connectivity spectrum captured via graph theoretical analyses, these measures are highly inter-correlated. For example, connectivity degree was highly correlated with efficiency within the control group for both structural (*r* = 0.71, *p* < 0.01) and functional connectivity (*r* = 0.96, *p* < 0.001). Subsequently, we compared each graph metric of both structural connectivity and task-related functional connectivity and found no significant correlations (as shown in Table 3). Weak correlations were found within the control group (0.1–0.3) and very weak to zero values of the correlations were observed within the TBI group (<0.1). In nodal characteristics, we found that there was only one significant positive correlation between functional and structural connectivity of the connectivity degree of the left superior medial frontal gyrus (Brodmann area 6, *r* = 0.63, *p* < 0.01, indicated in magenta in Figure 4). No other significant associations were observed between functional and structural connectivity. From these results, it is clear that there is no overall agreement between functional and structural connectivity within the switching network.

**Table 3:** **Results of the correlation analyses between graph metrics of structural connectivity and functional connectivity**.

**Graph metric**	**Functional connectivity (fMRI)**			
	**Control group**		**TBI group**	
**Structural connectivity (DTI)**	***r***	***p***	***r***	***p***
Strength	−0.119	0.661	0.121	0.643
Degree	−0.259	0.332	−0.029	0.913
Efficiency	−0.25	0.35	−0.075	0.775
Betweenness centrality	0.124	0.647	0.038	0.886

(Very) weak to none correlations were found within both groups.

### Control condition: FA-weighted graph analysis

An additional weighted graph analysis was performed in which the weight of the connections was represented by the FA value of the interconnecting WM tracts rather than the number of trajectories. Similar to the streamlines-weighted analysis, no significant group differences in structural connectivity were observed (all *p*'s > 0.05, see Table S1). Moreover, consistent with the results of the correlation analyses of the number of WM trajectories weighted analysis, very weak correlations were found between structural and functional connectivity for the FA-weighted analysis (see Table S2).

### Identification of hubs

To identify the hub regions, we calculated the betweenness centrality for each node of each subject's functional and structural network. Then, we calculated the mean betweenness centrality of each node by averaging across subjects for each group for each modality. For the functional connectivity, the identified hub nodes included the right dorsolateral prefrontal cortex, right insula lobe, and left dorsal premotor cortex for the TBI group (see yellow spheres in Figure 4). The hubs for the control group included only the right insula lobe. By contrast, for the structural connectivity, the bilateral subthalamic nuclei and right precuneus were identified as hub regions for both groups (indicated in cyan in Figure 4).

### Classification accuracy

Discriminant function analyses were performed on the behavioral data (switch accuracy as outcome variable) and connectivity degree (of each modality separately and in combination) to obtain a more effective and significant discrimination between the two groups. All models reached a Wilks' lambda of zero, indicating that their discriminatory power was sufficient enough to correctly classify most subjects. Classification accuracy for each model, that is, how well each model (structural or functional connectivity or combination) correctly identifies group membership, was calculated. The model based exclusively on one modality was less effective in distinguishing between good and worse performers on the LGT (functional: sensitivity: 42.9%; specificity: 70.6%, overall: 58.1%; structural: sensitivity: 28.6%; specificity: 76.5%, overall: 54.8%). The discriminatory power of the model based on the combination of the degree of functional and structural connectivity was slightly higher, achieving a sensitivity of 42.9% and specificity of 76.5% in the present sample (overall classification accuracy: 61.3%).

## Discussion

The present study examined the relation between structural and functional connectivity in patients with TBI from a graph theoretical network perspective. We found that connectivity matrices obtained using both diffusion MRI and task-related fMRI showed no agreement. In addition, we observed that by combining complementary information from the different imaging modalities the accuracy in prediction of switching performance is improved.

### Executive control deficits in TBI patients

The behavioral results showed that the information of the easy conditions (repeat and global trials) was processed faster than the information from the difficult conditions (switch and local trials). Moreover, controls outperformed TBI patients on the switching task. The healthy controls completed the executive control tasks more rapidly than the TBI patients and they presented higher accuracy rates during the LGT. These results are consistent with our previous studies (Caeyenberghs et al., 2012a; Leunissen et al., 2013a) suggesting that these switching deficits in TBI patients may be related to disruptions in the cortico-subcortical connectivity, limiting the ability to enforce efficient cognitive control over action.

### Group differences in connectivity

The topological architecture of the functional networks was significantly altered in patients with TBI. Specifically, an increase in connection strength was consistently observed in patients with TBI. Strength provides information on the variability of node local connectivity in the brain and tells whether brain nodes are all more or less connected to the same number of nodes. Strength is defined as the weighted variant of the degree and is the sum of all neighboring link weights (Rubinov and Sporns, 2010). Thus an increase in strength in TBI patients implies that their network connections are relatively denser than in controls. Increased functional brain connectivity has also been reported in our previous studies in TBI adults and children (Caeyenberghs et al., 2011, 2012a), in elderly (Heitger et al., 2013), and in patients with brain tumors (Bartolomei et al., 2006; Bosma et al., 2008). Even though it is intuitively appealing to suggest that higher connectivity strength in clinical groups may reflect functional compensation, it is clearly not the whole story. Strength is also thought to reflect neurodevelopmental exigencies of wiring cost minimization, and network topological feature optimization (Bullmore and Sporns, 2012). At the cellular level, it would be metabolically difficult in TBI to maintain an extremely high number of connections because this metabolism is simply not sustainable (Griffa et al., 2013). Thus, a higher connection strength can point to high levels of energetic cost, indicative of an overcharged network that is unbalanced in the transmission versus energy consumption trade-off, as recently observed in a longitudinal study in patients with TBI using resting-state magnetoencephalogram recordings (Castellanos et al., 2011).

The absence of group effects on graph metrics of structural connectivity tends to suggest that TBI does not strongly affect the structural connectivity or organization of the switching network. This result is not consistent with our previous study (Caeyenberghs et al., 2012b,c), in which the nodes were defined using the automated anatomical labeling atlas (AAL, Tzourio-Mazoyer et al., 2002) covering the whole brain. However, Smith et al. (2011) suggested that a data-driven approach by defining networks based only on areas showing clear task-related activation is preferable to template-based approaches in order to minimize confounds and obtain a better picture on brain connectivity.

### Correlation between structural connectivity and task-related functional connectivity

To the best of our knowledge, DTI and task-related fMRI have not been combined in a graph theoretical approach in TBI patients. Our results show no significant association between graph metrics of structural and functional connectivity. No significant correlations between each graph metric, including connectivity degree, connection strength, regional efficiency, and betweenness centrality, of both structural connectivity and task-related functional connectivity were found. Moreover, the hubs obtained using both techniques showed no agreement. The right dorsolateral prefrontal cortex, right insula lobe, and left dorsal premotor cortex presented as hubs in the functional connectivity. By contrast, the bilateral subthalamic nuclei and right precuneus acted as hub in both groups for the structural connectivity. In other words, the topological properties of brain functional networks cannot be solely accounted for by the properties of the underlying structural networks in this clinical group.

Although there are many studies investigating structural and functional connectivity in the same cohort of participants, most of these studies have employed a “univariate” approach, where each modality is analyzed separately. For example, Palacios et al. (2012) found a significant relationship between mean FA values of several WM tracts, including the inferior and superior longitudinal fasciculi, cingulum, uncinate, and corpus callosum, and functional activation scores for the default mode network and working memory network. Although a valuable contribution is made by the concurrent consideration given to distinct DTI data and (resting-state) fMRI data, a systematic framework of integrating them is required to achieve reasonable inferential power.

The number of studies *directly* comparing functional and structural connectivity is relatively small (for a review, see Damoiseaux and Greicius, 2009). Although these studies use slightly different functional and structural units (including the default network, a set of predefined anatomical regions, voxels, etc), they show largely convergent results, i.e., the strength of resting-state functional connectivity is positively correlated with structural connectivity strength (e.g., Hagmann et al., 2008, 2010; Skudlarski et al., 2008; van den Heuvel et al., 2008). Moreover, functional connectivity was also observed between regions when there is little or no structural connectivity, which most likely indicates functional correlations mediated by indirect structural connections (via one or more intermediate regions) (Greicius et al., 2009; Honey et al., 2009).

Both the mean connectivity matrices and the graph metrics at the nodal level show no similarity between DTI and fMRI estimates of graph metrics. This divergence between DTI and task-related connectivity may help us to understand the biological substrates of changes. Increased functional connectivity in the absence of reduction of structural connectivity would point to impaired network nodes that fail to utilize existing neuronal connections effectively. TBI might cause natural reorganization of functional connectivity giving rise to the decoupling between the two aspects of brain connectivity. While both methods assess particular aspects of brain connectivity, combining complementary information from the different imaging modalities can improve accuracy in prediction of behavioral deficits. Our results indicate that the multimodality classification approach yields significant improvement in accuracy over using each modality independently. The classification accuracy obtained by the combination is 61.3%, which is an increase of at least 3.2% from the single modality-based methods.

## Limitations and conclusions

The number of WM trajectories was used as a weight value for structural connectivity. Other definitions of edge weight for structural connectivity, such as FA, mean diffusivity, level of myelination, and the number of fibers have previously been used (e.g., Gong et al., 2009; Li et al., 2009; Hagmann et al., 2010; van den Heuvel et al., 2010; Vaessen et al., 2012). Currently, no consensus prevails which weighting factor is the most representative measure of structural connectivity in the construction of the graphs. To test the robustness of our results, we also constructed networks weighted by FA values (see Supplemental Material). The results of those networks were comparable with those of the presented WM networks (the number of WM trajectories and binary).

Moreover, in this study, we employed a deterministic streamline tractography (Basser et al., 2000; Mori and van Zijl, 2002) to define the edges of the structural network. This is by far the most widely applied tractography method in clinical research, mainly for its simplicity, robustness and speed (Cheng et al., 2012; Griffa et al., 2013). Nevertheless, deterministic tractography is known to be particularly sensitive to noise, and the tensor model is unable to recover multiple diffusion orientations in single voxels, making it impossible to reconstruct tracts passing through brain regions with complex fiber architecture, also referred to as “crossing fibers” (Tournier et al., 2011; Jeurissen et al., 2013). Graph theoretical analyses in clinical populations would surely benefit from the use of more advanced reconstruction and tractography techniques, such as diffusion spectrum magnetic resonance imaging (DSI) (Wedeen et al., 2005, 2008) or high angular resolution diffusion imaging (HARDI) with Q-ball reconstruction of multiple fiber orientations (Tuch, 2004; Hess et al., 2006; Jeurissen et al., 2011).

As the methodologies for measuring structural and functional connectivity improve and their complementarity strengths are applied in parallel, we expect important advances in our prognostic capacities for degree of brain injury. Even though our results should be interpreted with caution, to our knowledge, this is the first report combining measures of altered functional and structural connectivity of the switching network to elucidate the mechanisms responsible for cognitive deficits after brain injury.

## Funding

This research was supported by the Interuniversity Attraction Poles Programme initiated by the Belgian Science Policy Office (P7/11). Additional funding was obtained by the Research Fund KU Leuven (OT/11/071) and FWO Vlaanderen (G.0482.10, G.A114.11, G.0483.10; G.0721.12).

### Conflict of interest statement

The authors declare that the research was conducted in the absence of any commercial or financial relationships that could be construed as a potential conflict of interest.

## Supplementary material

Table S1 **Graph metrics of the switching network of the FA-weighted control analysis, mean, and standard error for both groups**.

Table S2 **Results of the correlation analyses between graph metrics of structural connectivity and functional connectivity**. (Very) weak to absent correlations were found within each of both groups.
