# Supplementary material for: Topological correlations of structural and functional networks in patients with traumatic brain injury
Source: Front Hum Neurosci. 2013 Nov 5;7:726. doi: 10.3389/fnhum.2013.00726 (PMC3817367; doi:10.3389/fnhum.2013.00726)
Supplement: Table S1 — Graph metrics of the switching network of the FA-weighted control analysis, mean, and standard error for both groups. [file DataSheet1.PDF]

Topological correlations of structural and functional networks in patients with traumatic brain injury

K. Caeyenberghs[1,2], A. Leemans[3], I. Leunissen[4], K. Michiels[5] & S.P. Swinnen[4]

[1]Department of Physical Therapy and Motor Rehabilitation, Faculty of Medicine and Health sciences, University of Ghent, Belgium

[2]Department of Movement and Sport Sciences, Faculty of Medicine and Health sciences, University of Ghent, Belgium

[3]Image Sciences Institute, University Medical Center Utrecht, Utrecht, The Netherlands

[4]Movement Control and Neuroplasticity Research Group , Group Biomedical Sciences, KU Leuven, Belgium

[5]Department of Physical Medicine and Rehabilitation, University Hospital, Leuven Campus Pellenberg, Belgium

Supplemental data

Table S1. Graph metrics of the switching network of the FA-weighted control analysis, mean, and standard error for both groups.

	TBI group (N=17)		control group (N=16)			
	mean	SE	mean	SE	T	p
**graph metric**						
**structural connectivity (DTI) FA weighted**						
strength	0,781	0,039	0,818	0,035	0,714	0,48
efficiency	0,349	0,027	0,314	0,022	-0,978	0,335
betweenness centrality	3,561	0,55	3,744	0,818	0,188	0,852

Table S2. Results of the correlation analyses between graph metrics of structural connectivity and functional connectivity. (Very) weak to absent correlations were found within each of both groups.

**graph metric**	functional connectivity (fMRI)			
	control group		TBI group	
structural connectivity (DTI)	*r*	*P*	*r*	*P*
Strength	0,016	0,952	0,075	0,775
Efficiency	-0,332	0,209	-0,066	0,803
betweenness centrality	0,124	0,647	0,038	0,886
